# Early career interview: Aya Mousa

**DOI:** 10.4155/fsoa-2019-0010

**Published:** 2019-04-12

**Authors:** Aya Mousa

**Affiliations:** 1 Research Fellow, Monash Centre for Health Research and Implementation, School of Public Health and Preventive Medicine, Monash University, Melbourne, VIC 3168, Australia

Aya Mousa was one of the five finalists for the Future Science Early Career Research Award 2018. Read her interview to find out about her career, hopes for the future and advice to other early career researchers.

## Please tell us about your career history to date

I obtained a Bachelor of Health Sciences degree (first class Hons) from the University of Auckland in New Zealand in 2011. I graduated with the highest grade point averages in both my undergraduate and postgraduate degrees, receiving the Senior Scholar Award (undergraduate BHSc, 2011) and Honours Prize (postgraduate BHSc Hons, 2012). I then worked as a teaching associate in epidemiology, biostatistics and public health (2013), before relocating to Australia on a scholarship to pursue a PhD in clinical sciences exploring the role of vitamin D in cardiometabolic diseases. My PhD was conferred in April 2018 from Monash University (Melbourne, Australia), after which I received an early career biomedical research fellowship from the National Health and Medical Research Council (NHMRC) of Australia commencing in 2019. Despite completing my PhD less than 1 year ago, I have authored more than 30 publications in distinguished journals. My research experience, coupled with collaborations I have formed with clinical leaders and experts has allowed me to possess a unique combination of skills across the research continuum (biomedical, epidemiological, clinical, public health and translation) that I will utilize and build on in my career moving forward. Currently, I am employed as an academic research fellow at Monash University where I am supervising PhD and Honours students and building my independent research team investigating interventions for the prevention of cardiometabolic diseases.

## What made you choose a career in your field?

Cardiometabolic and chronic diseases are major causes of mortality and morbidity worldwide and we are in dire need of simple and cost-effective solutions that can address this epidemic on a global scale. Vitamin D deficiency is also highly prevalent, affecting more than 1 billion people worldwide, primarily due to increased sedentary indoor lifestyles and limited sun exposure due to fear of skin cancer.

I first became interested in health research during my time at the University of Auckland in New Zealand, where I studied and taught epidemiology, biostatistics, research methods and public health for 4 years. I then started my PhD in Australia where I conducted extensive research exploring the role of vitamin D in diabetes, pregnancy and heart disease. During this time, I became interested in the field of clinical and prevention research, particularly in relation to simple nutritional interventions for preventing cardiometabolic diseases. It was during my PhD that I built on previous knowledge through formal coursework in advanced biostatistics and chronic disease epidemiology. I also enjoyed gaining practical clinical research skills by conducting a clinical trial from start to finish, as well as by performing epidemiological studies, large-scale evidence syntheses and fieldwork and translational activities.

Through this work, I realized for the first time the importance of medical research and its potential implications for human health. Together, these experiences prompted my interest in furthering my career in biomedical and clinical research with a focus on nutrition, metabolism and cardiometabolic diseases.

## Describe the main highlights of your career so far

I am truly blessed to have had a number of highlights during my research career thus far, including:

- Writing and publishing my research, including leading and/or being involved in over 30 papers in reputable journals;
- Receiving monetary support to be able to test my research questions and hypotheses. This includes securing over AUD $500,000 in competitive funding including as chief investigator on two young investigator project grants, an early career networking project grant and an early mid career research collaborative seed grant;
- Being invited to discuss my vitamin D research on a national radio station as well as having online news articles published about my work in vitamin D and inflammation;
- Receiving recognition through a number of awards during my academic career including the Senior Scholar Award, Honours Prize, Australian Postgraduate Award and 1st Prize Endocrinology Clinical Research Award, as well as the Premier's Award for Health and Medical Research (Finalist in the Clinical Research category – only five selected from across the state of Victoria). After submitting my thesis, I received two highly competitive postdoctoral fellowships to commence my postdoctoral research;
- Attending and presenting my research at multiple conferences including at two leading international diabetes congresses (American Diabetes Association; International Diabetes Federation);
- I have had the pleasure of teaching undergraduate students and supervising two PhD students and one Honours student so far, which is probably the greatest highlight of my career as I enjoy training future generations of researchers.

## Describe the most difficult challenge you have faced & how you overcame it

A difficult challenge often experienced by early career researchers (ECRs) is the transition toward becoming an independent researcher and moving your research beyond the benchtop such that it can be directly translated to improved patient care.

As an ECR, often it is possible to feel lost as to where your work fits in the bigger picture and how you can truly make a difference to the health of patients and communities in a real-world setting. I experienced this challenge as I moved from being a PhD student to becoming an independent ECR and it forced me to think beyond ‘track-records’ and ‘numbers of publications’ to focus on the more important aspect of research, that is, how to make a real impact on the health and well-being of mankind.

I started to formulate strategies to overcome this challenge and I decided to utilize an approach where I can generate and test my own research questions in a way which made them translatable and simple to scale up. This often means thinking of translation at the very start of the research process and then designing and refining your research accordingly. I now also ensure that I collaborate with other researchers and health professionals with different areas of expertise in order to consciously foster collaboration and promote an interdisciplinary approach to my work. I believe that this process has helped me design research which bridges the gap between benchtop and bedside and, by considering the translation aspect at the design stage and throughout the execution of my projects, I believe this will help me deliver research with direct impact for population health.

## How do you feel you have impacted your field?

In my short research career thus far, I feel I have made an important impact to the field of disease prevention research and that my work has provided new knowledge and unraveled novel pathways by which vitamin D may improve metabolic health. My research has addressed several knowledge gaps and identified new avenues to guide future research in this area. I was able to show that vitamin D is unlikely to be beneficial for the prevention of diabetes on a population scale but may reduce underlying risk factors such as inflammation. I have now started examining the role of vitamin D in inflammation in more detail and I have started exploring whether genetic variations related to vitamin D metabolism may play a role, such that perhaps subgroups of populations may benefit more than others. These findings may have important clinical implications for the treatment of vitamin D deficiency as well as for treating diseases associated with inflammation. I aim to continue impacting on this field by testing and validating other interventions for the prevention and treatment of cardiometabolic diseases in the hopes that my research will one day lead to meaningful discoveries and improved health outcomes.

## What are your aims for the future?

My aims for the future are:

- To contribute to knowledge and to make meaningful discoveries and share these discoveries for the greater good;
- To never stop questioning and to continue to search for truth, and never shy away from it, regardless of whether this truth agrees with my initial theories;
- To be meticulous in my methods and to work in partnership and build on the learnings of those who came before me;
- To continually and relentlessly seek knowledge, build my skills and further my understanding into how we as a society, scientific and otherwise, can improve health outcomes on a global scale and prevent chronic and debilitating diseases; and
- To teach, both formally and through leading by example, future generations of researchers and scientists, such that the knowledge and skills passed down to me can be carried forward for years to come.

## What advice do you have for early career researchers looking to take the next step in their career?

Being an early career researcher is not an easy feat! We dive head-first into a career where we need to develop a professional identity and research independence while competing for grants, teaching and supervising students, and dealing with increased administrative duties, all the while maintaining our research output. My advice to early career researchers is twofold; first, surround yourselves with the right people. That is, people who will take the time to support you, who will foster your growth and development, who will provide you with the opportunities you need to succeed and most of all, who will not take advantage of your hard work and diligence. Second, look for hidden opportunities in everything. Say yes, often. You may get overwhelmed and some jobs may seem daunting or pointless, but there are always opportunities, even if they are not immediately apparent. These opportunities may be as simple as collaborating with new colleagues or teams, demonstrating your skills or learning new ones, or simply getting your name out there and building a ‘go-getter’ reputation. As early career researchers, we often need to be mentored and provided with opportunities to excel, and being a ‘yes’ person goes a long way in ensuring that those opportunities keep coming.

